# The Root Hair Assay Facilitates the Use of Genetic and Pharmacological Tools in Order to Dissect Multiple Signalling Pathways That Lead to Programmed Cell Death

**DOI:** 10.1371/journal.pone.0094898

**Published:** 2014-04-22

**Authors:** Joanna Kacprzyk, Aoife Devine, Paul F. McCabe

**Affiliations:** School of Biology and Environmental Science, University College Dublin, Dublin, Ireland; University of Nottingham, United Kingdom

## Abstract

The activation of programmed cell death (PCD) is often a result of complex signalling pathways whose relationship and intersection are not well understood. We recently described a PCD root hair assay and proposed that it could be used to rapidly screen genetic or pharmacological modulators of PCD. To further assess the applicability of the root hair assay for studying multiple signalling pathways leading to PCD activation we have investigated the crosstalk between salicylic acid, autophagy and apoptosis-like PCD (AL-PCD) in *Arabidopsis thaliana*. The root hair assay was used to determine rates of AL-PCD induced by a panel of cell death inducing treatments in wild type plants treated with chemical modulators of salicylic acid synthesis or autophagy, and in genetic lines defective in autophagy or salicylic acid signalling. The assay demonstrated that PCD induced by exogenous salicylic acid or fumonisin B1 displayed a requirement for salicylic acid signalling and was partially dependent on the salicylic acid signal transducer NPR1. Autophagy deficiency resulted in an increase in the rates of AL-PCD induced by salicylic acid and fumonisin B1, but not by gibberellic acid or abiotic stress. The phenylalanine ammonia lyase-dependent salicylic acid synthesis pathway contributed only to death induced by salicylic acid and fumonisin B1. 3-Methyladenine, which is commonly used as an inhibitor of autophagy, appeared to influence PCD induction in all treatments suggesting a possible secondary, non-autophagic, effect on a core component of the plant PCD pathway. The results suggest that salicylic acid signalling is negatively regulated by autophagy during salicylic acid and mycotoxin-induced AL-PCD. However, this crosstalk does not appear to be directly involved in PCD induced by gibberellic acid or abiotic stress. This study demonstrates that the root hair assay is an effective tool for relatively rapid investigation of complex signalling pathways leading to the activation of PCD.

## Introduction

Programmed cell death is a crucial component of development and defence responses [1]. In plant cells apoptosis-like programmed cell death (AL-PCD) is characterized by cytoplasm condensation, leaving a visible gap between the cell wall and the plasma membrane and resulting in specific corpse morphology [1], [2], a feature that has been a useful tool in quantifying rates of AL-PCD in plant suspension cultures. Recently, we reported that a similar corpse morphology can be scored by a root hair assay, a novel technique for quantitative determination of AL-PCD rates in plants *in vivo* [3]. The root hair assay is a relatively fast and straightforward method based on observation of dying root hair morphology. We investigated whether the root hair assay can be used for dissecting multiple signalling pathways leading to AL-PCD by studying the putative crosstalk between SA, autophagy and AL-PCD in *Arabidopsis thaliana*.

Salicylic acid (SA) is involved in the regulation of a number of processes throughout the plant life cycle. It has been shown to play a role in growth, senescence and seed production, and the regulation of plant development relies on a coordinated crosstalk between SA and other phytohormones [4]. It has been established that SA has a role in plant immunity related programmed cell death (PCD) although that role is not yet fully understood, for example, following pathogen infection endogenous levels of SA increase in tissues surrounding cells that undergo hypersensitive response (HR) PCD [5]. SA has been also shown to be required for development of systemic acquired resistance [6], [7] and exogenously applied SA has been found to induce the expression of several pathogenesis-related proteins in plants [8], [9]. Genetic studies employing salicylic acid induction-deficient mutants provide further evidence of a role for SA during HR [10], [11].

The role of SA during HR often involves activating mechanisms that can increase reactive oxygen species (ROS) generation [10]–[14], and ROS have been implicated in triggering PCD [15]. Exogenously applied SA induced programmed cell death *in vitro* in Arabidopsis [16], rice [17] and tomato [18] cell suspension cultures and also promoted superoxide induced programmed cell death in Arabidopsis leaves [19]. It also induced cell death when applied to whole plants of lsd1 mutants [12] and in RPW8 enhanced transcription lines kept under conditions nonpermissive to spontaneous lesions formation [14].

Autophagy is a routine recycling pathway, occurring at a basal level in all growing plant cells. However, it has been also reported to play a role in starvation, development and defence responses to pathogens [20]. Investigations into the role of autophagy was facilitated by the isolation of more than thirty autophagy related (ATG) genes in yeast [20], [21] and several of the plant homologues were identified on the basis of sequence comparisons [20], [22], [23]. Numerous studies of autophagy deficient mutant phenotypes have been performed to unravel the roles of autophagy in plants. The basic role of autophagy during starvation, as a pro-survival mechanism ensuring efficient nutrient distribution, has been confirmed by the early senescence and high sensitivity to nutrient stress phenotypes observed in autophagy defective mutant plants [24], [25], [23], [26], [27]. However, under optimal growth conditions, autophagy defective Arabidopsis plants [25], [23], [26] undergo normal developmental processes such as embryogenesis, germination, shoot and root formation/elongation, flowering and seed production. In plants, autophagic vesicle formation, and their subsequent delivery to the vacuole, requires the conjugation of ATG8 and ATG12 protein tags to phosphatidylethanolamine and the ATG5 protein respectively. ATG7 is the enzyme required to initiate ligation of both ATG8 and ATG12 [26]. In this study, we used mutant plants atg7 and atg5, which have been shown to be autophagy deficient, as they fail to accumulate GFP-ATG8-labeled vesicles in the vacuolar lumen. They also display an early senescence and hypersensitivity to nutrient limiting conditions phenotype which is characteristic of autophagy deficient plants [26], [25], [28].

Recently, it has been suggested that during pathogen induced PCD there exists crosstalk between SA signalling and autophagy. Yoshimoto *et al*., [29] observed increased senescence and HR related PCD in autophagy defective atg mutants and linked it with increased SA accumulation in these genotypes. Indeed atg phenotypes were found to be SA signalling dependent, as the excessive cell death was not observed in the *atg5 sid2* and *atg5 npr1* double mutants, characterized by the reduction of SA biosynthesis and blocked SA signalling respectively. Moreover, application of a SA agonist induced a senescence/cell death phenotype in SA-deficient *atg* mutants but not in *atg npr1* plants, suggesting that the cell death phenotypes in the *atg* mutants are dependent on NON-EXPRESSOR OF PATHOGENESIS-RELATED GENES1 (NPR1). The authors proposed that, in addition to its role in nutrient recycling, plant autophagy negatively regulates senescence and HR-related PCD by operating a negative feedback loop modulating SA signalling [29].

In plants, SA acid has been proposed to be generated via two distinct enzymatic pathways. The phenylalanine ammonia lyase (PAL) pathway involves conversion of chorismate-derived l-phenylalanine into SA via coumaric acid and a series of enzymatic reactions initially catalyzed by PAL, whereas the second isochorismate synthase (ICS) pathway involves conversion of chorismate into SA via isochorismate in a two-step process catalysed by ICS and isochorismate pyruvate lyase (IPL) (reviewed by [30]). Although the majority of SA production appears to be dependent on the ICS pathway, a double mutation of two Arabidopsis isochorismate genes ics1/ics2 results in a plant that contains residual SA confirming that this pathway is not the only source of SA production [31]. SA can also be produced via the PAL pathway, indeed suppression of PAL in tobacco resulted in fourfold decrease of SA levels in plants treated with tobacco mosaic virus [32]. Moreover, the PAL gene was observed to be rapidly induced in response to infection with *Pseudomonas syringae*, wounding [33], infection with *Botrytis cinerea* [34] or treatment with the bacterial elicitor flagellin [35]. The PAL inhibitor, 2-aminoindan-2-phosphonic acid (AIP) was shown to attenuate, but not fully suppress, SA accumulation in response to the hypersensitive response elicitor, arachidonic acid [36]. Taking into consideration that silencing or disruption of ICS results in a drastic reduction of pathogen- or UV-induced SA accumulation, while silencing or inhibition of PAL also has a major impact on pathogen-induced SA accumulation, it is possible that SA synthesis in plants relies on intermediates from both pathways [37].

In this paper we have used the root hair assay, and a panel of stress treatments, to ascertain if SA signalling and/or autophagy are core components of the regulatory mechanism that operates during AL-PCD regardless of the induction stimuli. Both a genetic approach (investigation of AL-PCD rates in mutant/transgenic lines defective in autophagy or SA signalling) and a pharmacological approach (application of modulators of SA synthesis or autophagy) were used. We found that SA is a potent inducer of AL-PCD in Arabidopsis root hairs. Death was preceded by early mitochondrial swelling and cell corpses displayed the protoplast retraction away from the cell wall that is a characteristic hallmark feature of AL-PCD. Our data distinguished between AL-PCD induced by SA or FB1 and AL-PCD induced by abiotic stress or gibberellic acid. For example, the genetic impairment of SA signalling (npr1-1 mutant), or accumulation (NahG transgene), lowered rates of AL-PCD induced by SA or FB1 but not the AL-PCD induced by gibberellic acid or various abiotic stresses. Inhibition of autophagy by wortmannin or autophagy deficiency in atg5 and atg7 plants resulted in an increase of AL-PCD rates induced by SA and FB1 suggesting a pro-survival role of autophagy during cell death induced by SA and FB1. This is presumably due to negative control of SA signalling by autophagy. We also show that SA synthesised by the PAL pathway, rather than the ICS pathway, contributes to the cell death response induced by SA and FB1. SA signalling and autophagy did not appear to be directly involved in the control of cell death induced by gibberellic acid or abiotic stress, suggesting that SA or autophagy are not fundamental regulators of AL-PCD but can act upstream of cell death in certain situations. It needs to be highlighted that the application of the root hair assay yielded results that fit well with the current model of SA-autophagy-programmed cell death cross-talk, suggesting that this technique is indeed a promising tool for dissecting complex PCD regulatory pathways.

## Materials and Methods

All chemicals were purchased from Sigma (UK) unless otherwise stated.

### Plant material and growth conditions

Seeds of *Arabidopsis thaliana* Col-0 ecotype WT, npr1-1, NahG, sid2, atg5 and atg7 were sterilised for 20 min in 20% (V/V) commercial bleach (final concentration of NaOCl approximately 1%) followed by washing x 4 with sterile distilled water (SDW). Following sterilisation, seeds where plated in a single line on half-strength MS (basal salts, 2.15 g l^−1^) medium, 1% sucrose, 1.5% agar in 12×12 cm square Petri dishes and vernalized at 4°C for 1–3 days in the dark before being placed vertically under constant light (6 µmol m^−2^ s^−1^) at 22°C.

### Cell death induction

#### Heat treatment

Heat treatment was carried out in SDW using a Grant OLS200 waterbath set at 49°C, without shaking, for 10 min. Five day old seedlings were carefully transferred to wells of 24-well multiwell culture plates. Each well contained 1 ml of SDW. Plates were sealed with Leucopore tape and allowed to float in the waterbath for 10 min. Following heat treatment seedlings were returned to a constant temperature room at 22°C under constant illumination until scoring. Treatment at 49°C typically results in induction of between 30 to 70% AL-PCD in Arabidopsis root hairs within 24 hr.

#### NaCl treatment

Five day old *Arabidopsis thaliana* seedlings were incubated in 6 cm Petri dishes containing 5 ml of 100 mM NaCl solution for 5 min. Following treatment seedlings were transferred to 6 cm Petri dishes containing 5 ml of SDW and incubated in a constant temperature room at 22°C under constant illumination until scoring.

#### Gibberellic acid treatment

Five day old *Arabidopsis thaliana* seedlings were incubated in 6 cm Petri dishes containing 5 ml of 0.2 mM gibberellic acid (GA) solution for 30 min. Following treatment seedlings were transferred to 6 cm Petri dishes containing 5 ml of SDW and kept in constant light in a constant temperature room at 22°C until scoring.

#### Fumonisin B1 treatment

Five day old *Arabidopsis thaliana* seedlings were placed in wells of 24-well multiwell culture plates containing 1 ml of 50 µM fumonisin B1 (FB1, Cayman Chemicals, USA) aqueous solution. Following treatment seedlings were kept in constant light in a constant temperature room at 22°C until scoring.

#### SA treatment

Five day old *Arabidopsis thaliana* seedlings were placed in 6 cm Petri dishes containing 5 ml of 65 µM SA solution. The Petri dishes containing the seedlings were then incubated in a constant temperature room at 22°C under constant illumination until scoring.

#### H_2_O_2_ treatment

Five day old *Arabidopsis thaliana* seedlings were incubated in 6 cm Petri dishes containing 5 ml of 25 mM H_2_O_2_ solution for 5 min. Following treatment seedlings were transferred to 6 cm Petri dishes containing 5 ml of SDW and kept in constant light in a constant temperature room at 22°C until scoring.

### Wortmannin treatment

Five day old Col-0 seedlings were incubated in 7.5 µM wortmannin (A. G. Scientific, USA) solution in deionised water at 22°C, constant light for 6 hr prior to death-inducing treatment. Wortmannin stock (1 mM) was prepared in dimethyl sulfoxide (DMSO) and stored at −20°C. Solvent controls (0.075% DMSO v/v) were also prepared. Subsequent cell death inducing treatments and incubation prior to the root hair assay were also performed in the presence of 7.5 µM wortmannin/0.075% DMSO.

### 3-Methyladenine treatment

Five day old seedlings were incubated in 0.5 mM 3-methyladenine (3-MA) solution in deionised water at 22°C, constant light for 24 hr prior to death-inducing treatment. A 0.1 M 3-MA stock was prepared in deionised water. Prior to use, 3-MA stock was re-dissolved by heating. Cell death inducing treatments and incubation prior to the root hair assay were also performed in the presence of 0.5 mM 3-MA.

### AIP treatment

Col-0 seedlings were incubated in 40 µM AIP solution in SDW at 22°C, constant light for 2 hr prior to death-inducing treatment. Cell death inducing treatments and incubation prior to the root hair assay were also performed in the presence of 40 µM AIP.

### AL-PCD assay

The assay was performed as described by Hogg *et al*., [3]. Seedlings were incubated with the viability indicator, fluorescein diacetate (FDA). Only viable root hairs are able to cleave FDA to form fluorescein which, when excited by a wavelength of 485 nm, fluoresces green. Whole seedlings were stained in a 1 µg ml^−1^ solution of FDA on standard microscope slides and immediately examined under white light and fluorescent light. Root hairs that were positive for FDA staining were scored as alive. Root hairs negative for FDA staining were examined further and scored as either AL-PCD: having a condensed cell content and protoplast retracted away from the cell wall, or necrotic: having no retracted cytoplasm and therefore no distinguishable morphology compared to living cells under the light microscope. The percentage for each category was calculated as a percentage of the total number of roots hairs scored (typically ∼100) averaged over at least three replicates.

### Dye loading and confocal laser scanning microscopy

In order to investigate the effect of 3-MA on mitochondrial swelling during AL-PCD induction, seedlings were stained with the fluorescent dye Mito Tracker Green FM (MTG, Molecular Probes, Netherlands), which covalently binds to mitochondrial proteins regardless of the membrane potential [38] and can be used as the marker of mitochondrial masses. Seedlings were incubated in 70 nM MTG for 30 min at room temperature, in the dark and washed with distilled water directly prior to confocal imaging. Confocal imaging of mitochondria was performed with Olympus Fluoview FV1000 microscope. Specimens were examined using 60x immersion oil objective. MTG was excited with an argon laser (488 nm) and detected at 515 nm to 520 nm. The size of mitochondria was expressed as the average area of MTG signal. The approximate area of each individual MTG signal (mitochondrial cross section) was determined by manually measuring its shortest and longest diameter and calculating the area of the resulting ellipse on the basis of collected values. Typically, an average size of 10 mitochondria per root hair was recorded.

### Statistical analysis

Statistical Analysis Software (SAS, USA) and Microsoft Excel Analysis ToolPack add-in were used for statistical analysis of data.

## Results

### SA and mycotoxin induced AL-PCD is higher in autophagy mutants

**Figure 1:**
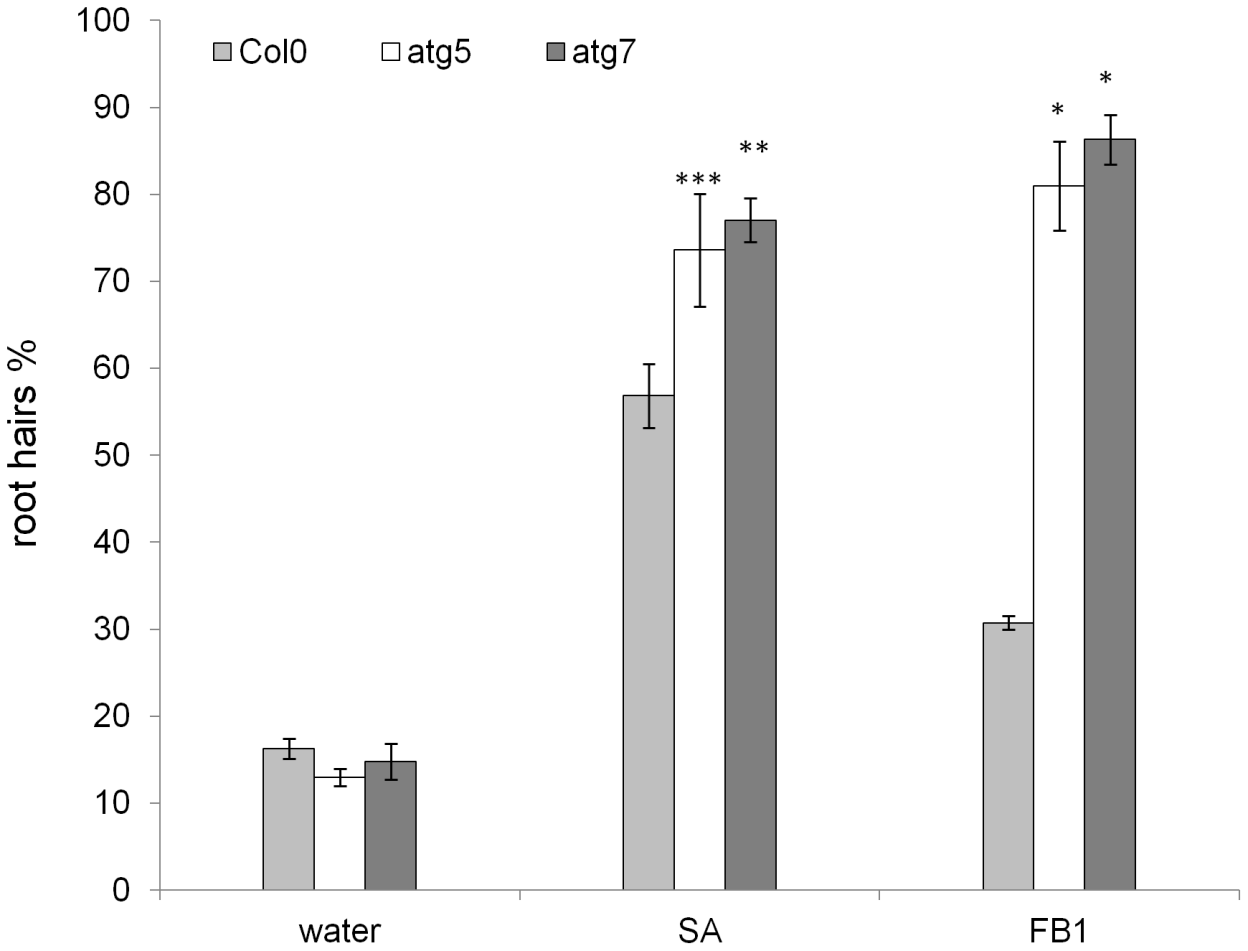
Autophagy deficiency results in an increase of SA and FB1 induced AL-PCD rates. Col-0, atg5 and atg7 seedlings were subjected to 65 µM SA treatment or 50 µM FB1 treatment. A root hair assay was performed 24 hr after the start of the treatment. Root hairs showing retraction and condensation of the cytoplasm and no FDA staining were deemed to have undergone AL-PCD (plotted). Root hairs showing neither FDA staining nor retraction of the cytoplasm were scored as necrotic (data not shown). There was no difference in rates of necrosis induced in Col-0, atg5 and atg7 seedlings. Viability rates in untreated Col-0, atg5 and atg7 seedlings were not statistically different. Presented values are means (n = 3) ±SEM. Effect of autophagy defective pathway was analyzed by student t-test: * p<0.01, ** p<0.05, ***p = 0.08. The experiment was repeated 3 times with similar results.

In order to study the crosstalk between SA signalling and autophagy, 5-day old seedlings of Col-0 and autophagy defective lines: atg5 and atg7, were subjected to 65 µM SA treatment. After 24 hr the root hair assay was performed and percentages of AL-PCD and total cell death (both AL-PCD and necrosis) were recorded. Both atg5 and atg7 autophagy defective lines had increased levels of AL-PCD compared to the wild type (Fig. 1), and this difference was statistically significant (atg7, p<0.05; atg5, p = 0.08). A similar increase of AL-PCD rates in autophagy defective lines was observed following 50 µM FB1 treatment (Fig. 1). This result is similar to findings of Lenz *et al*., (2011) [39] who reported that Arabidopsis atg genotypes developed spreading necrosis after treatment with fumonisin B1 or upon infection with the necrotrophic fungal pathogen, *Alternaria brassicicola*.

### Rates of AL-PCD induced by heat, NaCl, H_2_O_2_ and GA are unchanged in autophagy mutants

**Figure 2:**
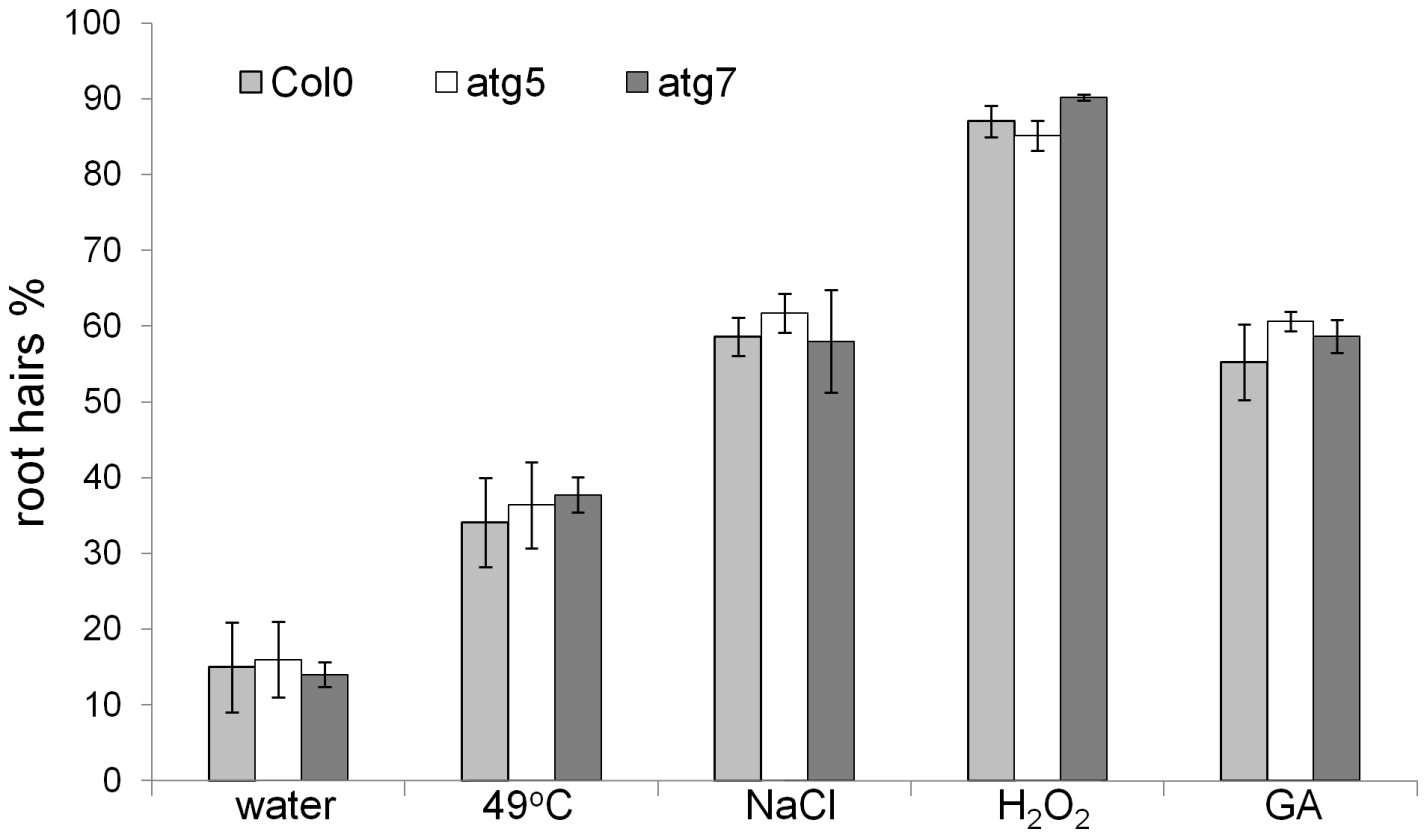
Autophagy deficiency does not affect AL-PCD induced by heat, NaCl, hydrogen peroxide or GA. Col-0, atg5 and atg7 seedlings were subjected to 49°C (10 min), 0.2 mM gibberellic acid (30 min), 25 mM hydrogen peroxide (5 min) and 100 mM sodium chloride (5 min) treatment. A root hair assay was performed 24 hr after the start of the treatment. Root hairs showing retraction and condensation of the cytoplasm and no FDA staining were deemed to have undergone AL-PCD (plotted). Root hairs showing neither FDA staining nor retraction of the cytoplasm were scored as necrotic (data not shown). There was no difference in rates of necrosis induced in Col-0, atg5 and atg7 seedlings. Viability rates in untreated Col-0, atg5 and atg7 seedlings were not statistically different. Presented values are means (n = 3) ±SEM. Statistical analysis (student t-test) shows no significant effect of autophagy deficiency on AL-PCD rates. The experiment was repeated 3 times with similar results.

In order to investigate the relationship between autophagy and abiotic stress-induced or gibberellic acid-induced AL-PCD, 5-day-old Col-0, atg5 and atg7 were subjected to series of treatments including: 49°C heat (10 min), 0.2 mM gibberellic acid (30 min), 25 mM hydrogen peroxide (5 min) and 100 mM sodium chloride (5 min) treatment. Twenty four hr following the treatments, the root hair assay was performed and percentages of AL-PCD and total cell death (both AL-PCD and necrosis) were recorded. No statistically significant difference was observed in the death response of wild type and autophagy defective plants (Fig. 2), suggesting that autophagy is not involved in regulation of cell death induced by these stresses.

### The autophagy inhibitor wortmannin leads to increases in AL-PCD following SA and mycotoxin treatment, but does not affect rates of AL-PCD following abiotic stress or gibberellic acid treatments

**Figure 3:**
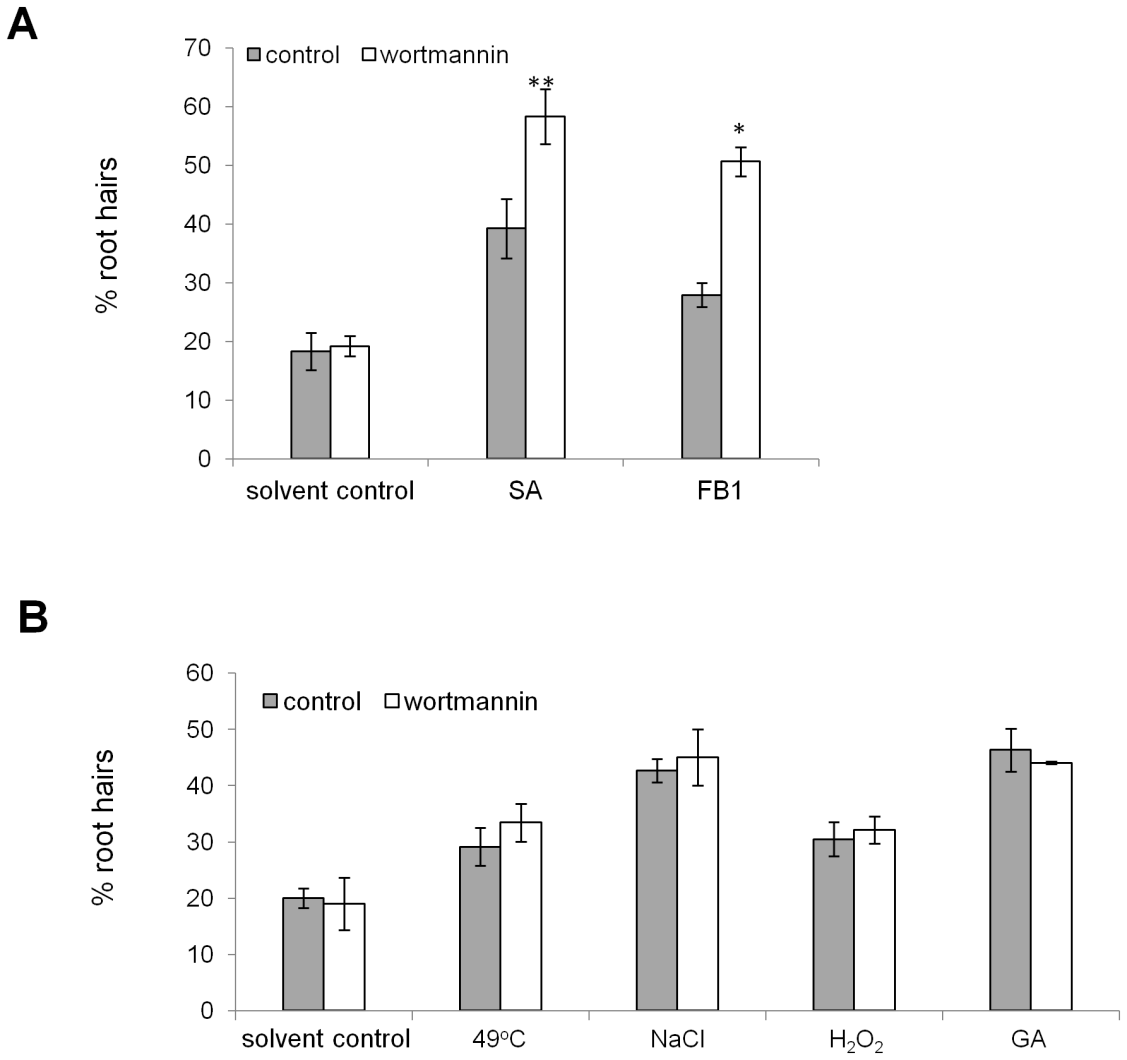
Wortmannin affects AL-PCD induced by SA and FB1 and not by abiotic stress and GA. Col-0 seedlings were pretreated with 7.5 µM wortmannin and cell death was induced by SA, FB1 (A) and abiotic stress: 49°C (10 min), 25 mM hydrogen peroxide (5 min) and 100 mM sodium chloride (5 min) treatment and 0.2 mM gibberellic acid (30 min) (B). A root hair assay was performed 24 hr after the start of the treatment and rates of necrosis (not shown) and AL-PCD (plotted) were recorded. There was no difference in rates of necrosis in control and wortmannin treated seedlings. Presented values are means (n = 3) ±SEM. Effect of wortmannin treatment was tested by student t-test: * p<0.01, ** p<0.05. The experiment was repeated 3 times with similar results.

Experiments with the autophagy inhibitor, wortmannin, were performed in order to further investigate whether increased sensitivity to SA and FB1 observed in atg5 and atg7 mutants was a specific result of a defective autophagy pathway rather than an unconnected mutant alteration. Phosphatidylinositol 3-kinase (PI3K) is important for membrane trafficking processes, which is an important factor in the autophagy pathway, both in the formation and fusion of vesicles [40]. Wortmannin inhibits PI3K and has been shown in animal cells to block autophagy at the sequestration step [41]. In plants wortmannin has been shown to significantly inhibit the accumulation of autophagosome-like structures in starved tobacco cells [42]. Col-0 seedlings were pretreated for 6 hr in 7.5 µM wortmannin or 0.075% v/v DMSO (solvent control) at 22°C under constant illumination. Death inducing treatments (0.2 mM gibberellic acid, 5 mM hydrogen peroxide, 100 mM sodium chloride, 65 µM SA, 50 µM FB1, 49°C heat shock), and post-treatment incubation, were also performed in the presence of wortmannin/DMSO. Twenty four hr following the treatments, the root hairs were scored and percentages of AL-PCD and total cell death (TCD, both AL-PCD and necrosis) were recorded. In order to exclude the effect of wortmannin's unspecific cellular toxicity, experimental repeats where control seedlings (not subjected to death inducing treatment) had TCD higher than 25% were rejected, which accounted for approximately one third of attempted experiments. Three or more independent, successful (no overwhelming toxic effect of wortmannin) experimental repeats were performed for each death-inducing treatment. As was found using seedlings of atg5 and atg7, FB1 and SA treatments induced a significantly higher percentage of AL-PCD and TCD in wortmannin treated seedlings than in control (Fig. 3A). Following heat, NaCl, hydrogen peroxide and gibberellic acid treatment (Fig. 3B), as was found in the study performed on autophagy defective mutants atg5 and atg7, no statistically significant difference was observed in the cell death response of wortmannin-treated and control seedlings.

### Treatment with the autophagy inhibitor 3-MA lowers rates of AL-PCD induced by all types of stress

**Figure 4:**
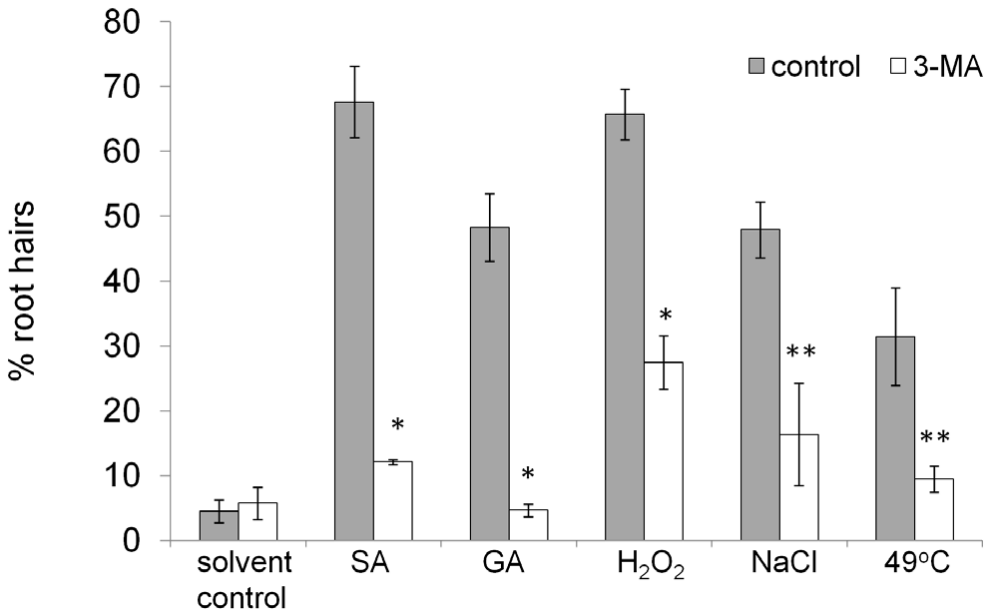
3-MA inhibits AL-PCD. Col-0 seedlings were pretreated with 0.5 mM 3-MA and cell death was induced by 65 µM SA, 0.2 mM gibberellic acid (30 min) and abiotic stress: 49°C (10 min), 25 mM hydrogen peroxide (5 min) and 100 mM sodium chloride (5 min) treatment. Root hair assay was performed after 6 hours following the start of stress treatment and rates of AL-PCD were plotted. Rates of necrosis remained unchanged as a result of 3-MA treatment (data not shown). 3-MA did not affect background (no stress treatment applied) levels of cell death. Presented values are means (n = 3) ±SEM. Effect of 3-MA treatment was tested by student t-test: * p<0.01, ** p<0.05. The experiment was repeated 3 times with similar results.

3-Methyladenine is a potent inhibitor of autophagy and it has been shown to inhibit starvation induced autophagy in tobacco culture cells [42], [43] and constitutive autophagy in Arabidopsis root tip cells [44]. In their study Inoue *et al*., [44], used 5 mM 3-MA to achieve partial inhibition of autophagy in the root tip cells of Arabidopsis seedlings, however, we found this level of 3-MA to induce significant rates of PCD in Arabidopsis root hairs. The highest non-toxic 3-MA concentration for the Arabidopsis root hairs was established (data not shown) to be 0.5 mM. Five-day old Arabidopsis seedlings were preincubated in 0.5 mM 3-MA for 24 hr prior to cell death inducing treatment at 22°C, constant light. Death inducing treatments (0.2 mM gibberellic acid, 25 mM hydrogen peroxide, 100 mM sodium chloride, 65 µM SA, 50 µM FB1, 49°C heat shock) and post-treatment incubation were also performed in the presence of 0.5 mM 3-MA. Six hr following the treatments, the root hairs were scored and percentages of AL-PCD, viability and necrosis were recorded. 3-MA significantly reduced the rates of AL-PCD induced by all tested treatments (Fig. 4). This effect was particularly dramatic in the case of phytohormone (salicylic and gibberellic acid) induced AL-PCD.

**Figure 5:**
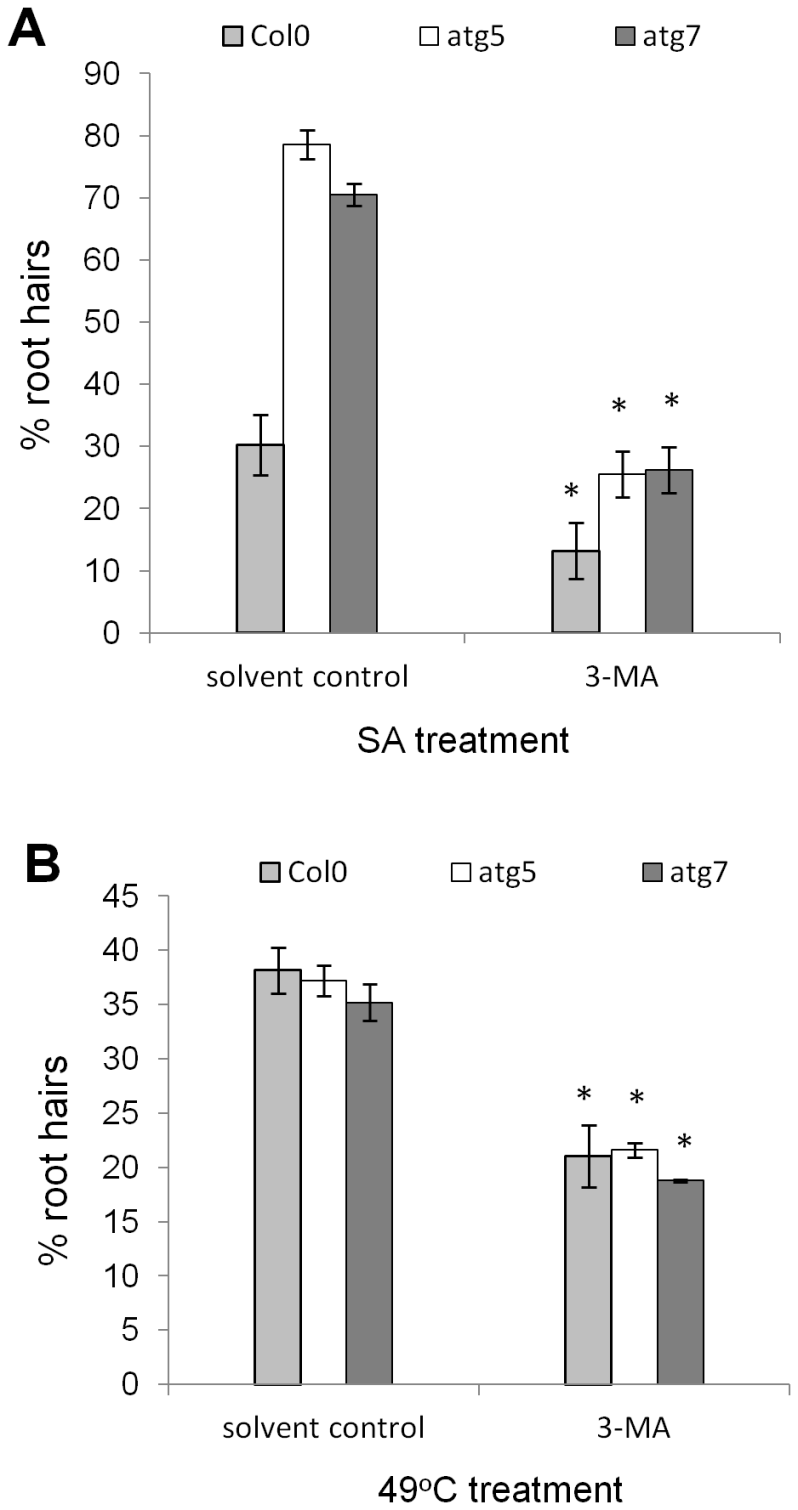
3-MA inhibits AL-PCD in autophagy defective atg5 and atg7 plants. Col-0, atg5 and atg7 seedlings were pretreated with 0.5 mM 3-MA and cell death was induced by 65 µM SA (A) and heat stress at 49°C (10 min) (B). Root hair assay was performed after 6 hours following the start of stress treatment and rates of AL-PCD were plotted. Rates of necrosis remained unchanged as a result of 3-MA treatment (data not shown). 3-MA did not affect background (no stress treatment applied) levels of cell death in Col-0, atg5 and atg7 seedlings. Presented values are means (n = 3) ±SEM. The effect of 3-MA treatment was tested by student t-test: * p<0.01. The experiment was repeated 3 times with similar results.

In order to account for the discrepancy between the pro-survival influence of 3-MA and the effect of autophagy deficiency on AL-PCD rates in atg5 and atg7 mutants, the following experiment was performed. 3-MA treatment was applied to autophagy defective plants. It was hypothesized that if the protective effect of 3-MA observed in Col-0 seedlings was the result of autophagy inhibition, then it should not be observed in atg5 and atg7 plants which are already autophagy deficient. 3-MA treatment was applied, as described above, on Col-0, atg5 and atg7 seedlings prior to cell death induction by heat (49°C) or SA. The root hair assay was scored 6 hr following the cell death induction. A protective effect of 3-MA was observed both in Col-0 and autophagy defective plants (Fig. 5). Remarkably, 3-MA inhibited most of AL-PCD induced in SA treated atg5 and atg7 plants. The results suggest that 3-MA, which is frequently used as an autophagy inhibitor may have significant secondary effects in plants.

In order to further investigate possible secondary effects of 3-MA, a study was performed where its effect on mitochondrial swelling, an early event during AL-PCD induction [45], was investigated. Col-0 seedlings were preincubated in 0.5 mM 3-MA or deionized water (control) for 24 hr prior to treatment with 65 µM SA (in presence or absence of 3-MA). After 1.5 hr of SA treatment, seedlings were stained in Mitotracker Green solution and analyzed by confocal microscopy. When the root hair assay was scored after 1.5 hr of SA treatment, only a small increase in levels of AL-PCD was observed (14.3% compared to 8.4% in control), however, after 24 hours levels of AL-PCD increased to 52.5% (compared to 8.7% in control). As previously found the 3-MA treatment blocked AL-PCD induced by SA.

**Figure 6:**
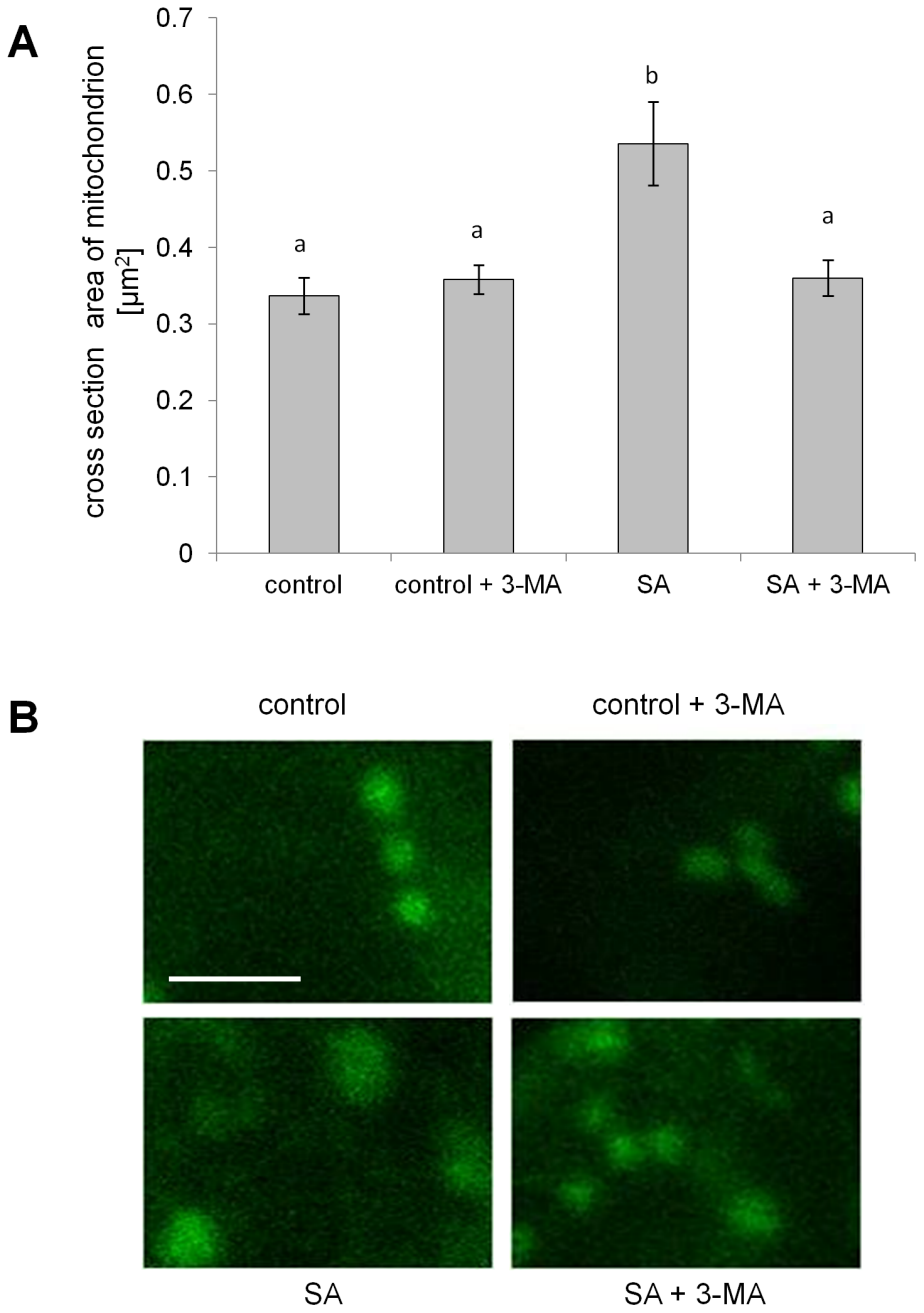
Mitochondrial swelling is induced by SA treatment and can be inhibited by 3-MA. Col-0 seedlings were preincubated in 0.5 mM 3-MA or distilled water (control) and subjected to 65 µM SA treatment (in presence or absence of 3-MA). After 1.5 hr of SA treatment, seedlings were stained with MTG and subjected to confocal imaging. **A**. The size of mitochondria expressed as the mean cross section area of individual MTG signal on the confocal microscopy image is presented. Typically, 3 seedlings, 2 root hairs per seedling were analyzed for each treatment and size of 10 mitochondria was measured per image (per root hair). Differences between the groups were evaluated by one way ANOVA and Tukey-Kramer multiple comparison test. Means with the same letter are not significantly different from each other. B. Typical images of root hair mitochondria, bar is 2.5 µm. The experiment was repeated twice with similar results.

Analysis of the mitochondrial size, defined as the average surface area of individual MTG signal, was performed on confocal images of root hairs. Mitochondria in root hairs treated with SA were characterized by a significant increase in size, indicating mitochondrial swelling, however, a similar effect was not observed in the presence of 3-MA (Fig. 6). This result may suggest that 3-MA affects the AL-PCD pathway upstream of the mitochondrial morphology transition, possibly by inhibiting opening of permeability transition pore.

### Inhibition of the PAL dependent SA synthesis pathway reduces AL-PCD induced by SA and FB1, but not cell death induced by abiotic stress and gibberellic acid

**Figure 7:**
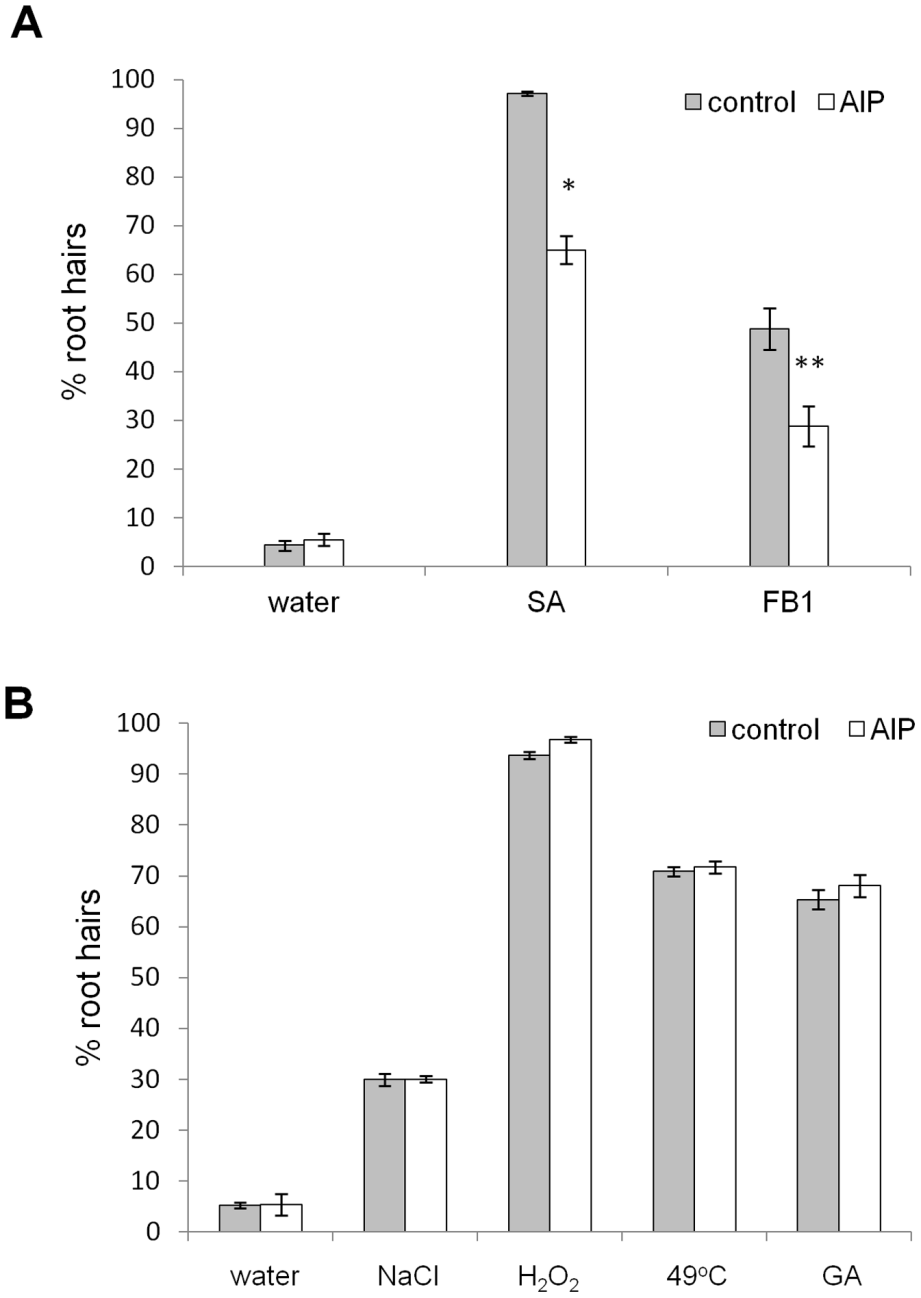
AIP inhibits AL-PCD induced by SA and FB1 but not by abiotic stress and GA. Col-0 seedlings were pretreated with 40 µM AIP and cell death was induced by 65 µM SA, 50 µM FB1, 0.2 mM gibberellic acid (30 min) and abiotic stress: 49°C (10 min), 25 mM hydrogen peroxide (5 min) and 100 mM sodium chloride (5 min) treatment. Root hair assay was performed after 24 hr following the start of stress treatment and rates of AL-PCD were plotted. Rates of necrosis remained unchanged as a result of AIP treatment (data not shown) and AIP did not affect background (no stress treatment applied) levels of cell death. Presented values are means (n = 3) ±SEM. Effect of AIP treatment was tested by student t-test: * p<0.01, ** p<0.05. The experiment was repeated 3 times with similar results.

In order to investigate the role of the phenylalanine ammonia lyase (PAL) dependent SA synthesis pathway in the death response of cells to a panel of stress treatments, a study with the SA antagonist, AIP, was performed. AIP is a highly specific inhibitor of PAL, and application of AIP has been shown to block SA accumulation in pathogen-infected Arabidopsis and elicitor-treated potato [46], [36]. Five day old Col-0 seedlings were preincubated in 40 µM AIP or deionised water (control) at 22°C, under constant illumination for 2 hr prior to death-inducing treatments. Death inducing treatments (15 mM hydrogen peroxide, 100 mM sodium chloride, 65 µM SA, 50 µM FB1, 49°C (heat shock) and post-treatment incubation were also performed in the presence of 40 µM AIP. Twenty four hr following the treatments, the root hair assay was scored and percentages of AL-PCD and total cell death, (TCD, both AL-PCD and necrosis) were recorded. AIP treatment significantly reduced levels of AL-PCD induced by SA and FB1 (Fig. 7A), however it did not affect death rates induced by hydrogen peroxide, NaCl or heat treatment (Fig. 7B).

### SA induced AL-PCD is affected in plants with disturbed SA signalling

In order to further investigate the role of SA in AL-PCD, experiments were performed on npr1-1, NahG and sid2 lines. NPR1 is a key regulator of systemic acquired resistance (SAR) and is essential for transduction of the SA signal and activation of pathogenesis related (PR) gene expression. Consequently, the npr1-1 mutant cannot respond to SA-mediated induction of PR genes [47]. Plants carrying the bacterial NahG transgene, which encodes salicylate hydroxylase, cannot accumulate a high level of SA [6]. The sid2 mutant is defective in the chloroplastic enzyme isochorismate synthase, a component of the ICS dependent SA synthesis pathway [48], [49].

**Figure 8:**
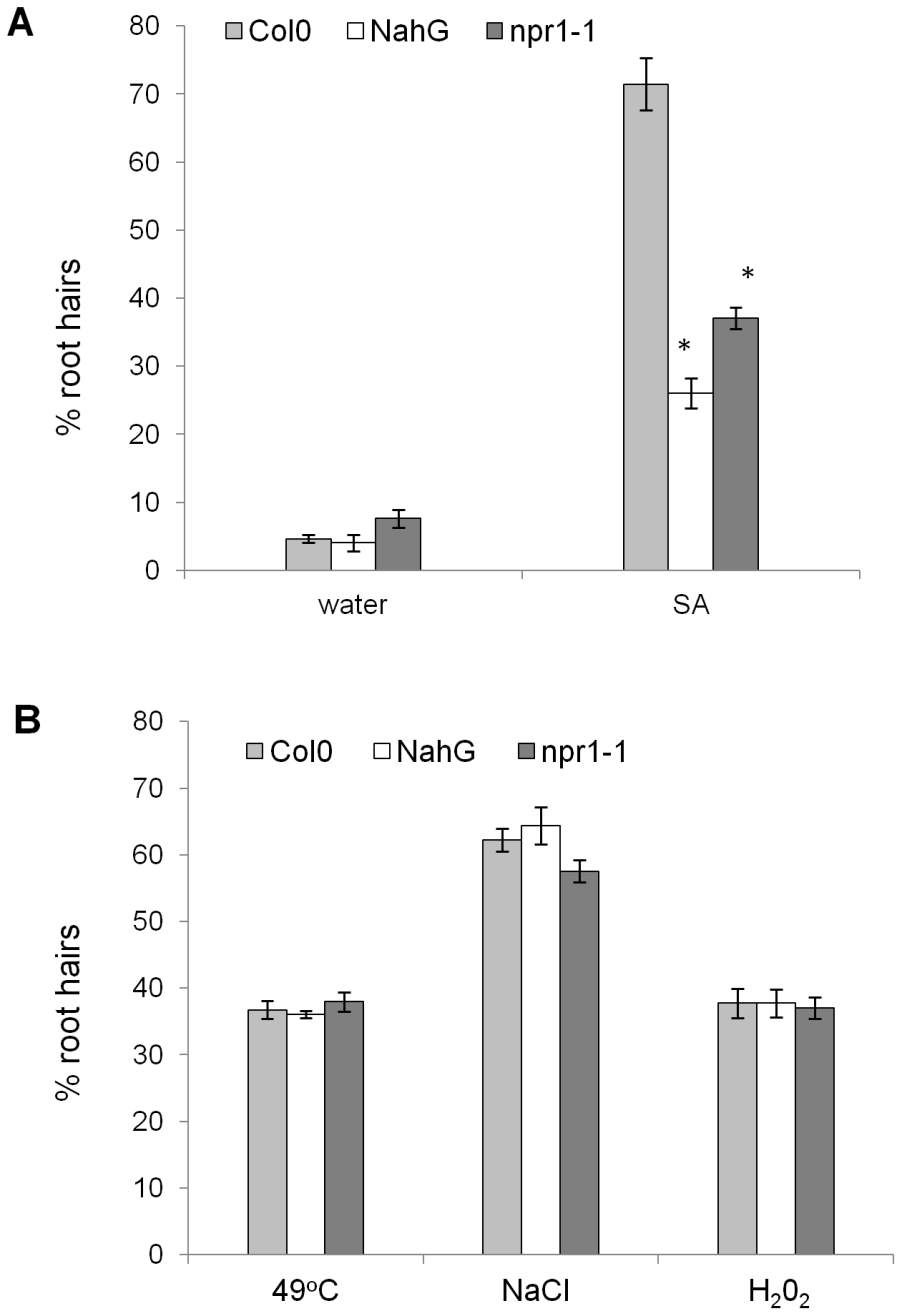
Disturbed SA acid signalling affects AL-PCD induced by SA and not by abiotic stress. Seedlings of Col-0 and disturbed SA signalling mutants: npr1-1 and NahG were subjected to cell death inducing treatments: 65 µM SA (A) and abiotic stress: 49°C (10 min), 15 mM hydrogen peroxide (5 min) and 100 mM sodium chloride (5 min) (B). Root hair assay was performed after 24 hours following the start of stress treatment and rates AL-PCD were plotted. Rates of necrosis were not significantly affected by death inducing treatments (data not shown). Presented values are means (n = 3) ±SEM. Difference in the rates of AL-PCD induced in Col-0 and NahG/npr1-1 seedlings was tested by student t-test: * p<0.01, ** p<0.05. The experiment was repeated 3 times with similar results.

Five day old seedlings of Col-0, npr1-1 and NahG were subjected to cell death inducing treatments (15 mM hydrogen peroxide, 100 mM sodium chloride, 65 µM SA, 50 µM FB1, 49°C heat shock) and 24 hr following the treatments, root hairs were scored and percentages of AL-PCD and total cell death, (TCD, both AL-PCD and necrosis) were recorded. SA induced AL-PCD was significantly lower in npr1-1 and NahG lines compared to Col-0 (Fig. 8A), however there was no difference in these lines response to abiotic stress compared to wild type plants (Fig. 8B). Reduction in the rates of FB1-induced AL-PCD in NahG and npr1-1 seedlings was also observed, however this effect was not always statistically significant (data not shown). Surprisingly, no statistically significant difference in rates of AL-PCD was observed in sid2 mutant background, regardless of the cell death inducing stimuli (data not included), suggesting that the isochorismate mediated pathway of SA synthesis is not required for cell death induced in the root hair system.

## Discussion

The root hair assay has proven to be a rapid and useful method for studying multiple signalling pathways leading to activation of AL-PCD in plants. This novel technique facilitated comparisons between cell deaths induced by range of stimuli *in vivo*. Furthermore, the root hair assay may have potential for differentiating between the signalling processes that lead to induction of PCD and the core activation components of the AL-PCD pathway.

### SA induced AL-PCD is negatively controlled by autophagy

In this study SA has been shown to be a potent inducer of AL-PCD in Arabidopsis root hairs. The characteristic AL-PCD morphology induced by SA was accompanied by early mitochondrial swelling, which has been shown to occur during plant programmed cell death in numerous studies, including Arabidopsis protoplasts following application of chemical or heat stimuli [45], UV-C treatment [50], as well as during PCD related senescence in *Medicago truncatula* cell suspension cultures [51]. The rate of SA-induced AL-PCD in Arabidopsis root hairs increased in autophagy defective atg5 and atg7 seedlings, supporting the view that SA signalling leading to cell death is negatively regulated by autophagy. This hypothesis was recently proposed by Yoshimoto *et al*., [29] who suggested that plant autophagy operates a novel negative feedback loop modulating SA signalling to negatively regulate senescence and immunity-related PCD. The death response induced by fumonisin B1 also increased in atg genotypes, this agrees with Lenz *et al*., [39] who observed spreading necrosis in the leaves of Arabidopsis atg genotypes after treatment with fumonisin B1. Therefore, the quantitive data obtained by scoring the cytoplamic retraction associated with AL-PCD, following SA or FB1 induced death, correlates well with published data obtained by observations of cell death lesion formation in the leaves of atg genotypes subjected to similar insults [29], [39]. The increases in FB1 induced cell death in autophagy defective plants may be connected with disruption of SA signalling. Fumonisin B1 is a fungal analog of sphinganine produced by the necrotrophic pathogen *Fusarium verticillioides*, which inhibits acyl-CoA-dependent ceramide synthase activity, thereby disrupting complex sphingolipid biosynthesis, resulting in an increase of free sphingoid base levels [52], [53]. The presence of FB1 during germination and seedling establishment in maize was recently demonstrated to induce programmed cell death associated with accumulation of SA acid [54]. Exogenous application of sphinganine also induced SA accumulation, suggesting that FB1 may facilitate *Fusarium verticillioides* colonization by activating the SA pathway manipulating cell death. In the light of existing evidence for crosstalk between FB1 and SA signalling, the increased rates of AL-PCD induced by FB1 treatment in atg seedlings may be explained by the absence of autophagic control of SA signalling [29], which results in increased SA accumulation in atg plants [29], [39]. However, rates of AL-PCD induced by gibberellic acid or short term intensive abiotic stress (heat –49°C, 10 min; NaCl –100 mM, 5 min; hydrogen peroxide −25 mM, 5 min) were unaffected in atg plants, suggesting no direct involvement of SA signalling in these cell death pathways.

Similar results were obtained by application of the autophagy inhibitor, wortmannin, which caused significantly increased rates of AL-PCD in response to SA and FB1 treatments, whereas levels of AL-PCD induced by other tested stresses were unchanged. In contrast application of another putative autophagy inhibitor, 3-MA, resulted in a strong cellular protective effect and inhibition of AL-PCD regardless of the cell death inducing stimuli. However, we found this significant pro-survival effect of 3-MA in autophagy defective atg5 and atg7 plants as well as in the wild type, which suggests that 3-MA's action was not exclusively autophagy-related. The pro-survival effect of 3-MA, independent of the cell death inducing stimuli, may indicate that it affects a core element of the AL-PCD pathway. Moreover, 3-MA was observed to inhibit mitochondrial swelling during SA induced AL-PCD, suggesting that it acts upstream of the mitochondrial morphology transition. 3-MA is a widely used autophagy inhibitor in animal and plant studies, which has been shown to selectively inhibit autophagic/lysosomal protein degradation in rat hepatocytes [55] as well as to block autophagy in plant cells [42], [43], [44]. Studies on rat hepatocytes demonstrated that 3-MA inhibits autophagic sequestration due to its inhibitory action on PI3K [41]. However, the specificity of 3-MA as an autophagy inhibitor has been questioned. For example, 3-MA has been shown to block both autophagy and apoptosis in sympathetic neurons, where the cytochrome *c* release from mitochondria and caspase activation was inhibited [56]. More insight into the potential mode of action of 3-MA in an animal system was provided by Xue *et al*., [57] who studied the influence of 3-MA on mitochondrial status *in vitro* on isolated rat liver mitochondria. 3-MA inhibited mitochondrial swelling and cytochrome *c* release induced by calcium chloride and phenylarsine oxide in both heart and liver mitochondria. It has been proposed that 3-MA might inhibit both autophagy and apoptosis by inhibiting mitochondrial permeability transition and thereby preserving mitochondrial function [57]. Evidence for a non autophagy related role of 3-MA was provided by Mizushima *et al*., [58], who reported that 3-MA can suppress proteolysis even in atg5-deficient embryonic stem cells, suggesting that its effects on protein degradation extend beyond its role in autophagy inhibition. Data presented in this study provides the first evidence of a dual role of 3-MA in a plant system, in addition to its previously reported role in inhibiting autophagy. This study has shown 3-MA can inhibit AL-PCD induced by a panel of treatments, presumably acting upstream of the mitochondrial morphology transition. This inhibition of AL-PCD by 3-MA was also consistently observed in autophagy defective knockouts of atg5 and atg7, providing convincing evidence that 3-MA effects are not exclusively autophagy-related. Therefore, it is advisable that the use of 3-MA as an autophagy inhibitor in plant systems should be undertaken with caution and a supplemental method of autophagy monitoring should be included.

Our data supports the published experimental evidence that autophagy operates a negative feedback loop modulating SA signalling to suppress cell death linked with a pathogen elicitor; however autophagy does not appear to significantly affect cell death responses induced by gibberellic acid or intensive, short term, abiotic stresses such as heat, salt or hydrogen peroxide. The latter is in contrast to previous reports suggesting that autophagy is required for salt stress tolerance in Arabidopsis [59]. Liu and colleagues [59] demonstrated that autophagy defective RNAi-AtATG18a plants are more sensitive to prolonged salt and drought stress. However, the role of autophagy under prolonged abiotic stress appears to be connected with removal of damaged proteins or organelles [60] rather than with the instant PCD response to stress.

### SA induced AL-PCD is mediated by the PAL dependent SA synthesis pathway and NPR1 signal transduction

Pathogen attack is often accompanied by increases in endogenous SA levels [5], [8], [9]. Additionally, application of exogenous SA, at levels that resulted in induction of cell death in a tobacco cell suspension culture, lead to a rapid and persistent increase in intracellular levels of SA of up to 10-fold higher than the externally applied concentration [61]. It can be hypothesised that an exogenous application of SA results in activation of a SA self amplifying feedback loop, which has been suggested to operate during pathogen defence [30]. We investigated whether AL-PCD induced by SA, or other treatments, is affected by a decrease in SA accumulation in NahG plants expressing salicylate hydroxylase or in SA insensitive npr1-1 mutant plants. It was found that rates of AL-PCD induced by SA were significantly lower in npr1-1 and NahG plants compared to controls. FB1 induced cell death was partially alleviated in the NahG background (data not shown), consistent with the findings by Asai *et al*., [62], who reported that protoplasts isolated from NahG plants are insensitive to FB1-induced cell death. Interestingly, rates of FB1 induced AL-PCD in root hairs of npr1-1 seedlings were also partially reduced, whereas data presented by Asai *et al*., [62] suggested that FB1-induced PCD in Arabidopsis protoplasts does not require the SA signal transmitter NPR1. However, Yoshimoto *et al*., [29] demonstrated that the early senescence phenotype, and excessive immunity related PCD, in atg mutants are dependent on the SA signal transducer NPR1. Rates of AL-PCD induced by gibberellic acid treatment or abiotic stress were not affected in npr1-1 and NahG plants, suggesting that any potential role of SA signalling in those types of death is not significant. It needs to be highlighted that there exists a number of contradictory reports about altered abiotic stress response phenotypes of SA insensitive mutants. For example, NahG transgenic Arabidopsis was less sensitive to prolonged salt and methyl viologen induced oxidative stress [63], however NahG potato plants were more sensitive to treatment with methyl viologen [64]. It is crucial to distinguish between the role of SA in prolonged stress tolerance and in the more rapid AL-PCD response to stress, which was the subject of this investigation.

SA has been proposed to be synthesized in plants via two distinct pathways: the ICS pathway and the PAL pathway. Both of these pathways originate from chorismate, however, to date neither biosynthetic route has been fully defined (reviewed by [65]). This study found that root hairs of Arabidopsis plants, pretreated with the PAL inhibitor, AIP, exhibited a small, but significant, suppression in levels of AL-PCD induced by SA or FB1, whereas gibberellic acid/abiotic stress induced death levels remained unchanged. Moreover, the rise in sphinganine levels caused by FB1 temporarily coincided with the induction of PAL transcript in maize [66] and the sphingolipid elicitor was also shown to induce mitogen activated protein kinase, which was required for PAL gene induction [67]. Therefore this data suggests that the PAL pathway does contribute to signalling involved in the induction of PCD by SA or FB1 in Arabidopsis root hairs. The rates of AL-PCD were not significantly affected in the sid2 mutant background, regardless of the cell death inducing stimuli. This is surprising, as it was previously suggested that the SA pool potentiating PCD may be derived from isochorismate synthesised by SID2, as the sid2 mutant is impaired in SA accumulation following pathogen inoculation [10], and there are examples of the sid2 mutation rescuing spontaneous cell death phenotypes [68], [69]. However, our findings are consistent with the results of Kadono *et al*., [70] who reported that ozone induced cell death, which shares similarities with the hypersensitive response observed in plant-pathogen interactions [71], [72], [73] was reduced in suspension culture in NahG and npr1 plants background but not in sid2. It can be concluded that SA synthesis via the ICS pathway does not seem to be required for AL-PCD induction in Arabidopsis root hairs.

### The root hair assay is a useful tool for identification of core components of the AL-PCD signalling pathway

**Figure 9:**
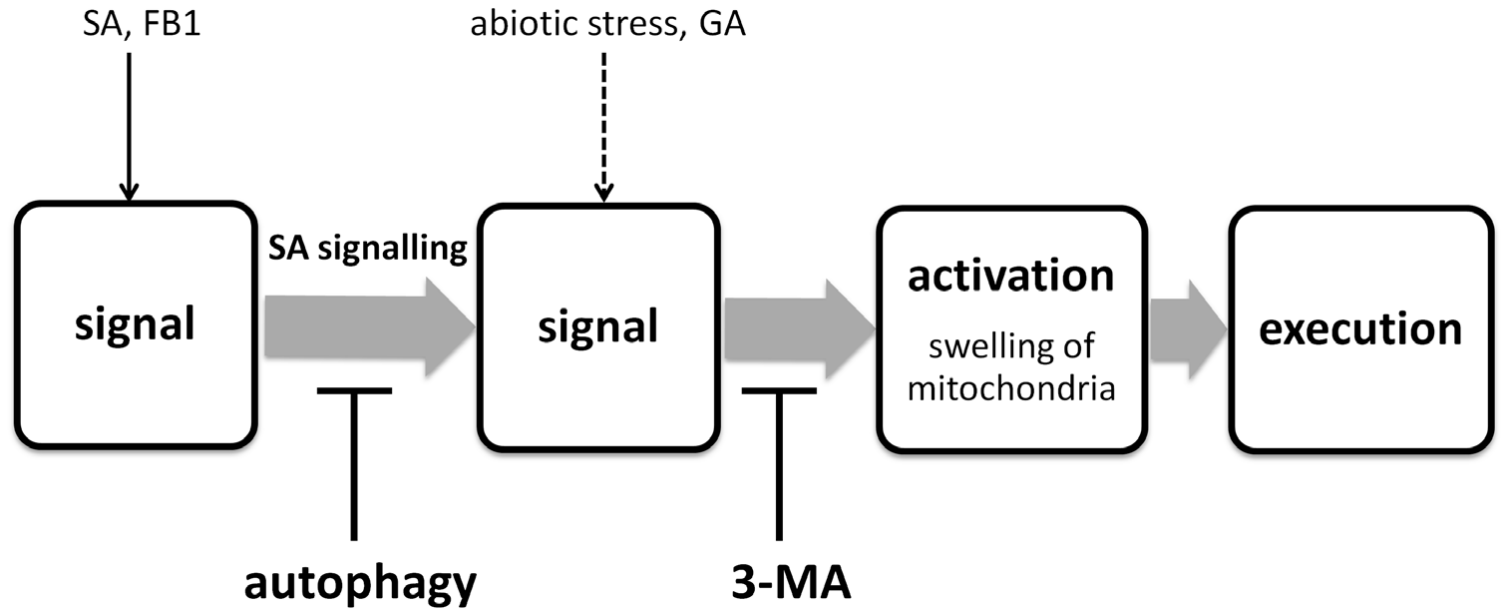
Model of SA, autophagy and abiotic stress induced signalling pathways leading to AL-PCD activation. 3-MA inhibits a core element of the AL-PCD pathway – either mitochondrial swelling or a component localized upstream of mitochondrial swelling - and therefore it effectively blocks AL-PCD regardless of cell death inducing stimuli. Autophagy and SA signalling crosstalk is a part of signalling pathway specific to cell death induced by SA or FB1 and therefore pharmacological or genetic manipulations of these signalling components do not affect levels of PCD induced by heat and GA acid. Potentially, there are cell death inducer specific components of the signalling pathway leading to cell death activation for abiotic stress and GA induced PCDs (dotted line).

Our results suggest that AL-PCD induced by SA and the mycotoxin FB1 have a requirement for SA signalling and is partially dependent on the SA signal transducer NPR1. Additionally, SA signalling appears to be negatively regulated by autophagy. Therefore, a pro-survival role for autophagy during SA and pathogen elicitor induced AL-PCD, which has been previously proposed for immunity-related PCD [29], is supported. During experiments to elucidate a potential role for autophagy in PCD, a secondary, non-autophagy specific role of the commonly used autophagy inhibitor 3-MA appeared to operate in plant cells, which previously has been only reported in animal cells [57]. The PAL dependent SA synthesis pathway has been shown to contribute to a cell death response induced by SA and FB1. However, SA signalling – autophagy crosstalk does not appear to be directly involved in cell death induced by gibberellic acid or abiotic stress. The root hair assay therefore proved to be a useful technique for studying multiple signalling pathways involved in activation of AL-PCD in plants. Additionally, the root hair assay can be used to differentiate between signalling effects specific to the cell death inducer and core components of AL-PCD signalling pathway (Fig 9). For example, autophagy deficiency in atg genotypes, or application of the autophagy inhibitor wortmannin, affected activation of AL-PCD induced only by SA and FB1. Similarly, these deaths were also dependent on SA signalling, whereas effects of other cell death inducers were not influenced by application of SA synthesis inhibitors or in SA signalling defective genotype. Therefore the cross talk between autophagy and SA signalling does not appear to directly activate the core AL-PCD machinery but a signalling pathway that may lead indirectly to activation of PCD. In contrast 3-MA blocked PCD regardless of cell death inducing stimuli, suggesting that 3-MA acts on a universal, core component of the AL-PCD pathway. Further analysis of the 3-MA effect with confocal microscopy suggested that this core component of the AL-PCD activation pathway is either upstream of mitochondrial swelling or is in fact the mitochondrial swelling itself (Fig 9).

Multiple signalling pathways can lead to the activation of PCD and these pathways can be activated in response to numerous intra- and extra-cellular stimuli. In order to increase our understanding of this complex signalling network it is essential to identify both core and cell death inducer-specific components of PCD signalling. As demonstrated in this study, the root hair assay is an invaluable, rapid, tool for testing the effects of multiple pharmacological and genetic modifications of cell biochemical pathways on *in vivo* rates of PCD induced by a range of stress stimuli. Therefore, this technique has the potential to contribute to unravelling the mechanisms involved in the induction, activation and destruction of plant cells undergoing PCD.

## References

[1] Kacprzyk J, Daly CT, McCabe PF (2011) The Botanical Dance of Death: Programmed Cell Death in Plants. In: Kader J-C, Delseny M, editors. Adv Bot Res vol.60. Academic Press. pp. 169–261.

[2] Reape TJ, McCabe PF (2013) Commentary: The cellular condensation of dying plant cells: Programmed retraction or necrotic collapse? Plant Sci 207: 135–139. 23602108 10.1016/j.plantsci.2013.03.001

[3] Hogg BV, Kacprzyk J, Molony E, O'Reilly C, Gallagher T, et al (2011) An *in vivo* root hair assay for determining rates of apoptotic-like programmed cell death in plants. Plant Methods 7: 45. 22165954 10.1186/1746-4811-7-45 PMC3266644

[4] Abreu ME, Munne-Bosch S (2009) Salicylic acid deficiency in NahG transgenic lines and sid2 mutants increases seed yield in the annual plant *Arabidopsis thaliana* . J Exp Bot 60: 1261–1271. 19188277 10.1093/jxb/ern363 PMC2657544

[5] Enyedi AJ, Yalpani N, Silverman P, Raskin I (1992) Localization, conjugation, and function of salicylic acid in tobacco during the hypersensitive reaction to tobacco mosaic virus. Proc Natl Acad Sci 89: 2480–2484. 1549613 10.1073/pnas.89.6.2480 PMC48682

[6] Gaffney T, Friedrich L, Vernooij B, Negrotto D, Nye G, et al (1993) Requirement of Salicylic Acid for the Induction of Systemic Acquired Resistance. Science 261: 754–756. 17757215 10.1126/science.261.5122.754

[7] Vernooij B, Friedrich L, Morse A, Reist R, Kolditz-Jawhar R, et al (1994) Salicylic acid is not the translocated signal responsible for inducing systemic acquired resistance but is required in signal transduction. Plant Cell 6: 959–965. 12244262 10.1105/tpc.6.7.959 PMC160492

[8] Malamy J, Carr JP, Klessig DF, Raskin I (1990) Salicylic acid: A likely endogenous signal in the resistance response of tobacco to viral infection. Science 250: 1002–1004. 17746925 10.1126/science.250.4983.1002

[9] Yalpani N, Silverman P, Wilson TM, Kleier DA, Raskin I (1991) Salicylic acid is a systemic signal and an inducer of pathogenesis-related proteins in virus-infected tobacco. Plant Cell 3: 809–818. 1820820 10.1105/tpc.3.8.809 PMC160048

[10] Nawrath C, Métraux J-P (1999) Salicylic acid induction deficient mutants of Arabidopsis express PR-2 and PR-5 and accumulate high levels of camalexin after pathogen inoculation. Plant Cell 11: 1393–1404. 10449575 10.1105/tpc.11.8.1393 PMC144293

[11] Mur LAJ, Bi Y-M, Darby RM, Firek S, Draper J (1997) Compromising early salicylic acid accumulation delays the hypersensitive response and increases viral dispersal during lesion establishment in TMV-infected tobacco. Plant J 12: 1113–1126. 9418052 10.1046/j.1365-313x.1997.12051113.x

[12] Dietrich RA, Delaney TP, Uknes SJ, Ward ER, Ryals JA, et al (1994) Arabidopsis mutants simulating disease resistance response. Cell 77: 565–577. 8187176 10.1016/0092-8674(94)90218-6

[13] Shirasu K, Nakajima H, Rajasekhar VK, Dixon RA, Lamb C (1997) Salicylic acid potentiates an agonist-dependent gain control that amplifies pathogen signals in the activation of defense mechanisms. Plant Cell 9: 261–270. 9061956 10.1105/tpc.9.2.261 PMC156916

[14] Xiao S, Brown S, Patrick E, Brearley C, Turner JG (2003) Enhanced transcription of the Arabidopsis disease resistance genes RPW8.1 and RPW8.2 via a salicylic acid–dependent amplification circuit is required for hypersensitive cell death. Plant Cell 15: 33–45. 12509520 10.1105/tpc.006940 PMC143449

[15] Van Breusegem F, Dat JF (2006) Reactive oxygen species in plant cell death. Plant Physiol 141: 384–390. 16760492 10.1104/pp.106.078295 PMC1475453

[16] García-Heredia J, Hervás M, De la Rosa M, Navarro J (2008) Acetylsalicylic acid induces programmed cell death in Arabidopsis cell cultures. Planta 228: 89–97. 18335236 10.1007/s00425-008-0721-5

[17] Matsumura H, Nirasawa S, Kiba A, Urasaki N, Saitoh H, et al (2003) Overexpression of Bax inhibitor suppresses the fungal elicitor-induced cell death in rice (*Oryza sativa* L.) cells. Plant J 33: 425–434. 12581301 10.1046/j.1365-313x.2003.01639.x

[18] Poór P, Kovács J, Szopkó D, Tari I (2012) Ethylene signaling in salt stress- and salicylic acid-induced programmed cell death in tomato suspension cells. Protoplasma 250: 1–12. 10.1007/s00709-012-0408-4 22535239

[19] Mazel A, Levine A (2001) Induction of cell death in Arabidopsis by superoxide in combination with salicylic acid or with protein synthesis inhibitors. Free Radical Biol Med 30: 98–106. 11134900 10.1016/s0891-5849(00)00452-4

[20] Bassham DC, Laporte M, Marty F, Moriyasu Y, Ohsumi Y, et al (2006) Autophagy in development and stress responses of plants. Autophagy 2: 2–11. 16874030 10.4161/auto.2092

[21] Klionsky DJ, Cregg JM, Dunn WA Jr, Emr SD, Sakai Y, et al (2003) A unified nomenclature for yeast autophagy-related genes. Dev Cell 5: 539–545. 14536056 10.1016/s1534-5807(03)00296-x

[22] Bassham DC (2007) Plant autophagy—more than a starvation response. Curr Opin in Plant Biol 10: 587–593. 17702643 10.1016/j.pbi.2007.06.006

[23] Hanaoka H, Noda T, Shirano Y, Kato T, Hayashi H, et al (2002) Leaf senescence and starvation-induced chlorosis are accelerated by the disruption of an Arabidopsis autophagy gene. Plant Physiol 129: 1181–1193. 12114572 10.1104/pp.011024 PMC166512

[24] Contento AL, Xiong Y, Bassham DC (2005) Visualization of autophagy in Arabidopsis using the fluorescent dye monodansylcadaverine and a GFP-AtATG8e fusion protein. Plant J 42: 598–608. 15860017 10.1111/j.1365-313X.2005.02396.x

[25] Doelling JH, Walker JM, Friedman EM, Thompson AR, Vierstra RD (2002) The APG8/12-activating enzyme APG7 is required for proper nutrient recycling and senescence in *Arabidopsis thaliana* . J Biol Chem 277: 33105–33114. 12070171 10.1074/jbc.M204630200

[26] Phillips AR, Suttangkakul A, Vierstra RD (2008) The ATG12-conjugating enzyme ATG10 is essential for autophagic vesicle formation in *Arabidopsis thaliana* . Genetics 178: 1339–1353. 18245858 10.1534/genetics.107.086199 PMC2278079

[27] Yoshimoto K, Hanaoka H, Sato S, Kato T, Tabata S, et al (2004) Processing of ATG8s, ubiquitin-like proteins, and their deconjugation by ATG4s are essential for plant autophagy. Plant Cell 16: 2967–2983. 15494556 10.1105/tpc.104.025395 PMC527192

[28] Thompson AR, Doelling JH, Suttangkakul A, Vierstra RD (2005) Autophagic nutrient recycling in Arabidopsis directed by the ATG8 and ATG12 conjugation pathways. Plant Physiol 138: 2097–2110. 16040659 10.1104/pp.105.060673 PMC1183398

[29] Yoshimoto K, Jikumaru Y, Kamiya Y, Kusano M, Consonni C, et al (2009) Autophagy negatively regulates cell death by controlling NPR1-dependent salicylic acid signaling during senescence and the innate immune response in Arabidopsis. Plant Cell 21: 2914–2927. 19773385 10.1105/tpc.109.068635 PMC2768913

[30] Vlot A, Dempsey D, Klessig D (2009) Salicylic acid, a multifaceted hormone to combat disease. Annu Rev Phytopathol 47: 177–206. 19400653 10.1146/annurev.phyto.050908.135202

[31] Garcion C, Lohmann A, Lamodière, Catinot J, Buchala A, et al (2008) Characterization and biological function of the ISOCHORISMATE SYNTHASE2 gene of Arabidopsis. Plant Physiol 147: 1279–1287. 18451262 10.1104/pp.108.119420 PMC2442540

[32] Pallas JA, Paiva NL, Lamb C, Dixon RA (1996) Tobacco plants epigenetically suppressed in phenylalanine ammonia-lyase expression do not develop systemic acquired resistance in response to infection by tobacco mosaic virus. Plant J 10: 281–293.

[33] Kohler A, Schwindling S, Conrath U (2002) Benzothiadiazole-induced priming for potentiated responses to pathogen infection, wounding, and infiltration of water into leaves requires the NPR1/NIM1 gene in Arabidopsis. Plant Physiol 128: 1046–1056. 11891259 10.1104/pp.010744 PMC152216

[34] Bézier A, Lambert B, Baillieul F (2002) study of defense-related gene expression in grapevine leaves and berries infected with *Botrytis cinerea* . Eur J Plant Pathol 108: 111–120.

[35] Tanaka N, Che FS, Watanabe N, Fujiwara S, Takayama S, et al (2003) Flagellin from an incompatible strain of *Acidovorax avenae* mediates H_2_O_2_ generation accompanying hypersensitive cell death and expression of PAL, Cht-1, and PBZ1, but not of LOX in rice. Mol Plant Microbe In 16: 422–428. 10.1094/MPMI.2003.16.5.422 12744513

[36] Coquoz J-L, Buchala A, Metraux J-P (1998) The biosynthesis of salicylic acid in potato plants. Plant Physiol 117: 1095–1101. 9662552 10.1104/pp.117.3.1095 PMC34925

[37] Chen Z, Zheng Z, Huang J, Lai Z, Fan B (2009) Biosynthesis of salicylic acid in plants. Plant Signal Behav 4: 493–496. 19816125 10.4161/psb.4.6.8392 PMC2688294

[38] Pendergrass W, Wolf N, Poot M (2004) Efficacy of MitoTracker Green™ and CMXrosamine to measure changes in mitochondrial membrane potentials in living cells and tissues. Cytometry Part A 61A: 162–169. 10.1002/cyto.a.20033 15382028

[39] Lenz HD, Haller E, Melzer E, Kober K, Wurster K, et al (2011) Autophagy differentially controls plant basal immunity to biotrophic and necrotrophic pathogens. Plant J 66: 818–830. 21332848 10.1111/j.1365-313X.2011.04546.x

[40] Shepherd PR, Reaves BJ, Davidson HW (1996) Phosphoinositide 3-kinases and membrane traffic. Trends Cell Biol 6: 92–97. 15157484 10.1016/0962-8924(96)80998-6

[41] Blommaart EFC, Krause U, Schellens JPM, Vreeling-Sindelárová H, Meijer AJ (1997) The phosphatidylinositol 3-Kinase inhibitors wortmannin and LY294002 inhibit autophagy in isolated rat hepatocytes. Eur J Biochem 243: 240–246. 9030745 10.1111/j.1432-1033.1997.0240a.x

[42] Takatsuka C, Inoue Y, Matsuoka K, Moriyasu Y (2004) 3-Methyladenine inhibits autophagy in tobacco culture cells under sucrose starvation conditions. Plant Cell Physiol 45: 265–274. 15047874 10.1093/pcp/pch031

[43] Inoue Y, Moriyasu Y (2006) Autophagy is not a main contributor to the degradation of phospholipids in tobacco cells cultured under sucrose starvation conditions. Plant Cell Physiol 47: 471–480. 16449232 10.1093/pcp/pcj013

[44] Inoue Y, Suzuki T, Hattori M, Yoshimoto K, Ohsumi Y, et al (2006) AtATG genes, homologs of yeast autophagy genes, are involved in constitutive autophagy in Arabidopsis root tip cells. Plant Cell Physiol 47: 1641–1652. 17085765 10.1093/pcp/pcl031

[45] Scott I, Logan DC (2008) Mitochondrial morphology transition is an early indicator of subsequent cell death in Arabidopsis. New Phytol 177: 90–101. 17986180 10.1111/j.1469-8137.2007.02255.x

[46] Mauch-Mani B, Slusarenko AJ (1996) Production of salicylic acid precursors is a major function of phenylalanine ammonia-lyase in the resistance of Arabidopsis to *Peronospora parasitica* . Plant Cell 8: 203–212. 12239383 10.1105/tpc.8.2.203 PMC161092

[47] Cao H, Bowling S, Gordon A, Dong X (1994) Characterization of an Arabidopsis mutant that is nonresponsive to inducers of systemic acquired resistance. Plant Cell 6: 1583–1592. 12244227 10.1105/tpc.6.11.1583 PMC160545

[48] Wildermuth MC, Dewdney J, Gang W, Ausubel FM (2001) Isochorismate synthase is required to synthesize salicylic acid for plant defence. Nature 414: 562–565. 11734859 10.1038/35107108

[49] Strawn MA, Marr SK, Inoue K, Inada N, Zubieta C, et al (2007) Arabidopsis isochorismate synthase functional in pathogen-induced salicylate biosynthesis exhibits properties consistent with a role in diverse stress responses. J Biol Chem 282: 5919–5933. 17190832 10.1074/jbc.M605193200

[50] Gao C, Xing D, Li L, Zhang L (2008) Implication of reactive oxygen species and mitochondrial dysfunction in the early stages of plant programmed cell death induced by ultraviolet-C overexposure. Planta 227: 755–767. 17972096 10.1007/s00425-007-0654-4

[51] Zottini M, Barizza E, Bastianelli F, Carimi F, Schiavo FL (2006) Growth and senescence of *Medicago truncatula* cultured cells are associated with characteristic mitochondrial morphology. New Phytol 172: 239–247. 16995912 10.1111/j.1469-8137.2006.01830.x

[52] Wang E, Norred WP, Bacon CW, Riley RT, Merrill AH Jr (1991) Inhibition of sphingolipid biosynthesis by fumonisins. Implications for diseases associated with Fusarium moniliforme. J Biol Chem 266: 14486–14490. 1860857

[53] Abbas HK, Tanaka T, Duke SO, Porter JK, Wray EM, et al (1994) Fumonisin- and AAL-toxin-induced disruption of sphingolipid metabolism with accumulation of free sphingoid bases. Plant Physiol 106: 1085–1093. 12232389 10.1104/pp.106.3.1085 PMC159634

[54] de la Torre-Hernandez EM, Rivas-San Vicente M, Greaves-Fernandez N, Cruz-Ortega R, Plasencia J (2010) Fumonisin B1 induces nuclease activation and salicylic acid accumulation through long-chain sphingoid base build-up in germinating maize. Physiol Mol Plant P 74: 337–345.

[55] Seglen PO, Gordon PB (1982) 3-Methyladenine: Specific inhibitor of autophagic/lysosomal protein degradation in isolated rat hepatocytes. Proc Natl Acad Sci 79: 1889–1892. 6952238 10.1073/pnas.79.6.1889 PMC346086

[56] Xue L, Fletcher GC, Tolkovsky AM (1999) Autophagy is activated by apoptotic signalling in sympathetic neurons: an alternative mechanism of death execution. Mol Cell Neurosci 14: 180–198. 10576889 10.1006/mcne.1999.0780

[57] Xue L, Borutaite V, Tolkovsky AM (2002) Inhibition of mitochondrial permeability transition and release of cytochrome c by anti-apoptotic nucleoside analogues. Biochem Pharma 64: 441–449. 10.1016/s0006-2952(02)01181-4 12147295

[58] Mizushima N, Yamamoto A, Hatano M, Kobayashi Y, Kabeya Y, et al (2001) Dissection of autophagosome formation using Apg5-deficient mouse embryonic stem cells. J Cell Biol 152: 657–668. 11266458 10.1083/jcb.152.4.657 PMC2195787

[59] Liu Y, Xiong Y, Bassham DC (2009) Autophagy is required for tolerance of drought and salt stress in plants. Autophagy 5: 954–963. 19587533 10.4161/auto.5.7.9290

[60] Liu Y, Bassham DC (2012) Autophagy: pathways for self-eating in plant cells. Annu Rev Plant Biol 63: 215–237. 22242963 10.1146/annurev-arplant-042811-105441

[61] Norman C, Howell KA, Millar AH, Whelan JM, Day DA (2004) Salicylic acid is an uncoupler and inhibitor of mitochondrial electron transport. Plant Physiol 134: 492–501. 14684840 10.1104/pp.103.031039 PMC316328

[62] Asai T, Stone JM, Heard JE, Kovtun Y, Yorgey P, et al (2000) Fumonisin B1–induced cell death in Arabidopsis protoplasts requires jasmonate-, ethylene-, and salicylate-dependent signaling pathways. Plant Cell 12: 1823–1836. 11041879 10.1105/tpc.12.10.1823 PMC149122

[63] Borsani O, Valpuesta V, Botella MA (2001) Evidence for a role of salicylic acid in the oxidative damage generated by NaCl and osmotic stress in Arabidopsis seedlings. Plant Physiol 126: 1024–1030. 11457953 10.1104/pp.126.3.1024 PMC116459

[64] Sanchez G, Gerhardt N, Siciliano F, Vojnov A, Malcuit I, et al (2010) Salicylic acid is involved in the Nb-mediated defense responses to potato virus X in *Solanum tuberosum* . Mol Plant Microbe In 23: 394–405. 10.1094/MPMI-23-4-0394 20192827

[65] Dempsey DMA, Vlot AC, Wildermuth MC, Klessig DF (2011) Salicylic acid biosynthesis and metabolism. Arabidopsis Book: e0156. 10.1199/tab.0156 PMC3268552 22303280

[66] Sánchez-Rangel D, Sánchez-Nieto S, Plasencia J (2012) Fumonisin B1, a toxin produced by *Fusarium verticillioides*, modulates maize β-1,3-glucanase activities involved in defense response. Planta 235: 965–978. 22120123 10.1007/s00425-011-1555-0

[67] Lieberherr D, Thao NP, Nakashima A, Umemura K, Kawasaki T, et al (2005) A sphingolipid elicitor-inducible mitogen-activated protein kinase is regulated by the small GTPase OsRac1 and heterotrimeric G-protein in rice. Plant Physiol 138: 1644–1652. 15951489 10.1104/pp.104.057414 PMC1176434

[68] Brodersen P, Malinovsky FG, Hématy K, Newman M-A, Mundy J (2005) The role of salicylic acid in the induction of cell death in Arabidopsis acd11. Plant Physiol 138: 1037–1045. 15923330 10.1104/pp.105.059303 PMC1150418

[69] Lu H, Salimian S, Gamelin E, Wang G, Fedorowski J, et al (2009) Genetic analysis of acd6-1 reveals complex defense networks and leads to identification of novel defense genes in Arabidopsis. Plant J 58: 401–412. 19144005 10.1111/j.1365-313X.2009.03791.x PMC2727925

[70] Kadono T, Tran D, Errakhi R, Hiramatsu T, Meimoun P, et al (2010) Increased anion channel activity is an unavoidable event in ozone-induced programmed cell death. PLoS ONE 5: e13373. 20967217 10.1371/journal.pone.0013373 PMC2954175

[71] Kangasjärvi J, Talvinen J, Utriainen M, Karjalainen R (1994) Plant defence systems induced by ozone. Plant Cell Environ 17: 783–794.

[72] Kangasjärvi J, Jaspers P, Kollist H (2005) Signalling and cell death in ozone-exposed plants. Plant Cell Environ 28: 1021–1036.

[73] Overmyer K, Brosché M, Pellinen R, Kuittinen T, Tuominen H, et al (2005) Ozone-induced programmed cell death in the Arabidopsis radical-induced cell death1 Mutant. Plant Physiol 137: 1092–1104. 15728341 10.1104/pp.104.055681 PMC1065409

